# First person – Daiana Almeida De Souza

**DOI:** 10.1242/bio.039313

**Published:** 2018-11-15

## Abstract

First Person is a series of interviews with the first authors of a selection of papers published in Biology Open, helping early-career researchers promote themselves alongside their papers. Daiana Almeida De Souza is first author on ‘Differences in the morphology, physiology and gene expression of honey bee queens and workers reared *in vitro* versus *in situ*’, published in BiO. Daiana is a postdoctoral researcher in the lab of David Tarpy at North Carolina State University, Raleigh, USA, investigating honey bees.


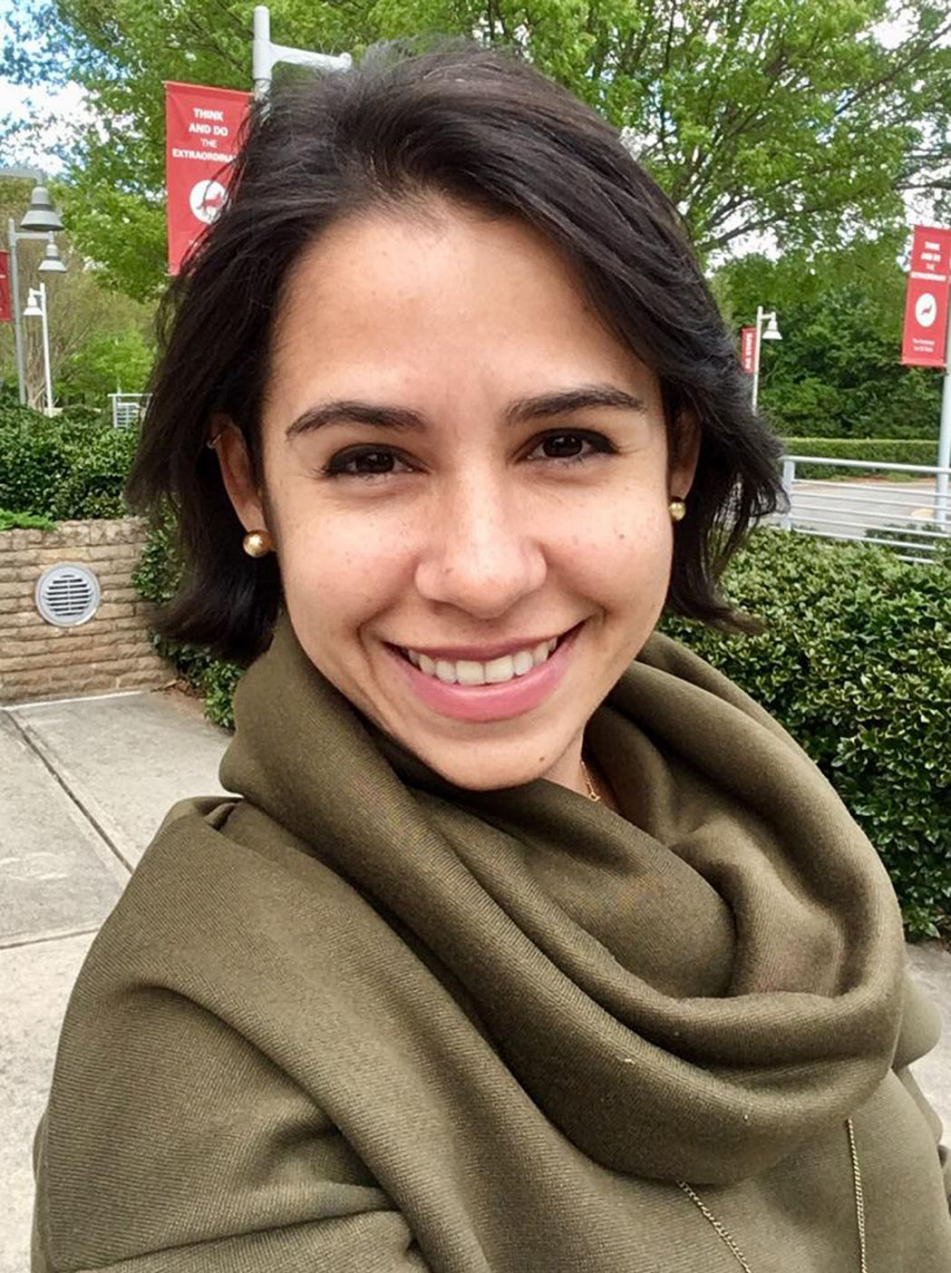


**Daiana Almeida De Souza**

**What is your scientific background and the general focus of your lab?**

Despite dedicating all my scientific career to honey bee (*Apis mellifera*) studies, my background could be considered a multi-faceted one. I have worked with honey bees since the beginning. I started with behavioral studies during my scientific initiation from my undergrad to my master's degree, where I had great experiences on this wonderful social insect's behavioral complexity, starting in my home country, Brazil. During my PhD, I began to work with honey bee queen quality, focusing on a genetic improvement program, looking for morphological features that would indicate better reproductive performance both physiologically, genetically and behaviorally. During this time, I noticed that the developmental biology trajectory of immature forms up to adulthood is much more important in how a future queen leads their colony than we were previously considering, due to the intricate caste determination process. This assertion led me to my first country outing to the U.S., where I worked in Robert Page's lab at Arizona State University, studying how the environment drives the fertility and reproductive quality of female bees during immature instar stages. This led me to David Tarpy's lab at North Carolina State University, where I'm currently working.

Tarpy's lab focuses on understanding the proximate and ultimate mechanisms of honey bee queen behavior and reproduction, in order to integrate a general understanding of bee biology to help improve overall colony health and productivity. My job there is to study the many morphological and physiological characteristics of honey bee queen quality, specifically investigating potential means of boosting queen quality through hormonal and nutritional augmentation during ontogenic development.

**How would you explain the main findings of your paper to non-scientific family and friends?**

The queen is the only individual within a colony of honey bees who is fully able to lay eggs. Her function in the colony ranges from egg production to the maintenance of colony order, which is reflected in the overall condition of her colony and, consequently, the productivity of the thousands of worker bees that make up a honey bee colony. By productivity, it's not only the honey and others bee products, but also the pollination activity that improves yields for thousands of crops. In this scenario my role is to find the best ways to improve the queen quality in order to improve the colony as a whole. This has led me to study biological features, such as morphology, physiology and genetics, which could potentially contribute to managing and improving the quality of the colony system.

“[…] external morphological traits cannot be fully translated into bee quality.”

**What are the potential implications of these results for your field of research?**

In this work we found that external morphological traits cannot be fully translated into bee quality. It's an interesting result since most of features we analyzed are commonly used as a bee quality reference. Moreover, our physiological and gene expression data suggest that protein, lipid and glucose metabolisms are regulated independently during the honey bee larval stage. That is helpful information for further investigations, especially in an ascending field such as the developmental biology applied to honey bee queen quality for beekeeping.

**Figure:**
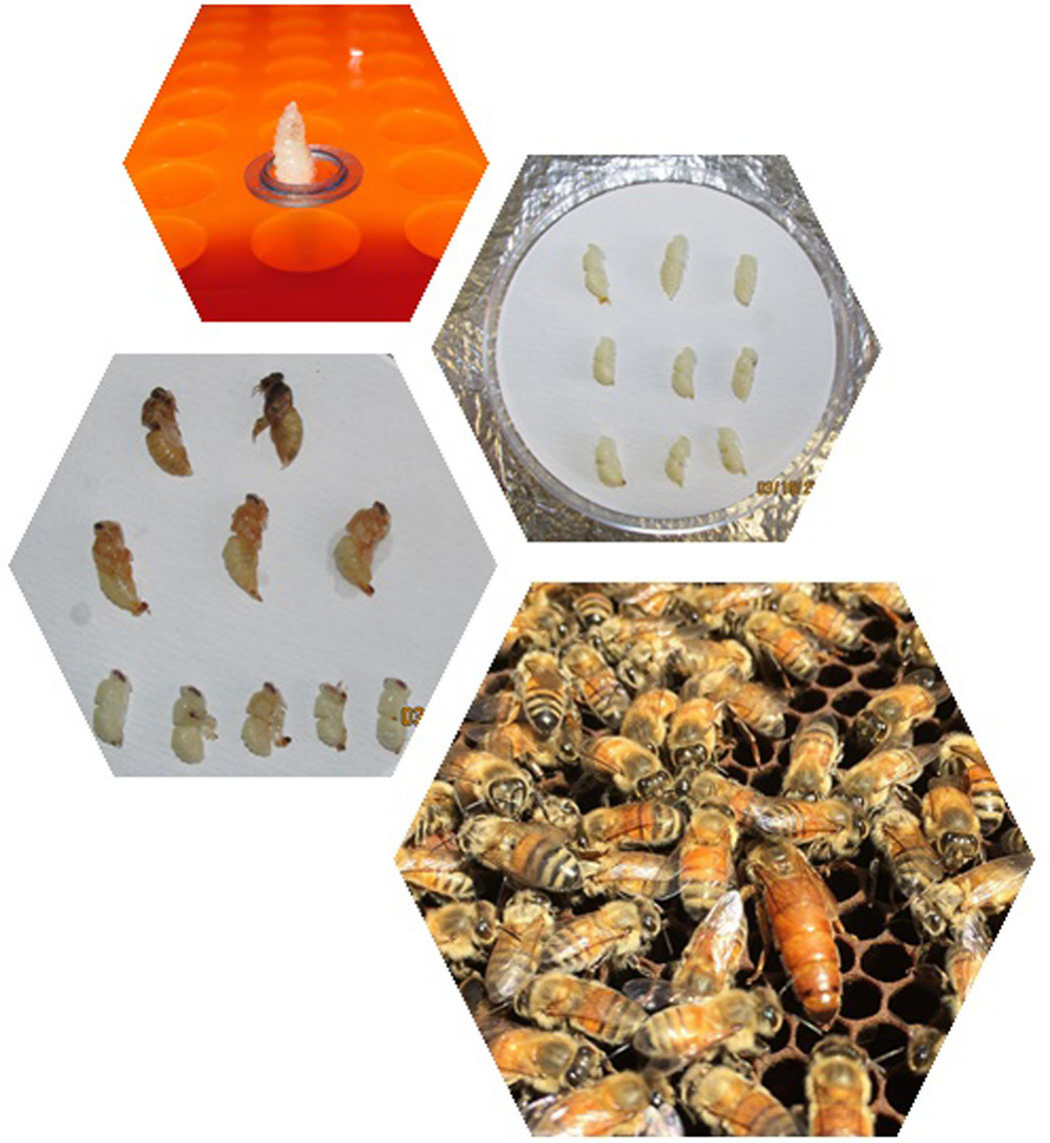
**Honey bees (*Apis mellifera*).**

**What has surprised you the most while conducting your research?**

It's general knowledge that the food quantity and quality drive the caste determination in honey bee females. However, when I did this work raising honey bees in an *in vitro* environment, I realized to my surprise that this conceptual dogma is not as pervasive as I thought. After doing some replication in which all larvae were raised in exactly the same food environment, I continued to have adults emerge presenting a degree of phenotypes from queens to workers. This shows us how the caste determination could be widely variable and how not just quality and quantity of food are involved in this adult phenotype determination.

**What, in your opinion, are some of the greatest achievements in your field and how has this influenced your research?**

The growth of epigenetic knowledge is greatly contributing to resolving some old issues and also clarifying how the social environment works by mediating changes in gene expression levels.

**What changes do you think could improve the professional lives of early-career scientists?**

Opportunity, and in some countries it should be considered as a real job. In some countries, such as Brazil, scientific research is not a career, which hugely reduces the opportunities to do science in these environments.

**What's next for you?**

Now that we know more about the intricate feeding dynamics that orchestrate reproductive traits of honey bee females, the next steps are to further investigate how it could be managed in way to improve the honey bee queen quality in practice. I've been working on ovary ontogenic development, and some physiology pathways involved in that in order to check the effects on further reproductive quality features that affect the colony as a whole.
